# Supplementary material for: Bacterial cell-size changes resulting from altering the relative expression of Min proteins
Source: Nat Commun. 2023 Sep 15;14:5710. doi: 10.1038/s41467-023-41487-0 (PMC10504268; doi:10.1038/s41467-023-41487-0)
Supplement: Supplementary file 1 — Supplementary Information [file 41467_2023_41487_MOESM1_ESM.pdf]

Supplementary Information

Bacterial cell-size changes resulting from altering the relative expression of Min proteins

Harsh Vashistha[1,♯,†], Joanna Jammal-Touma[1,♯], Kulveer Singh[2], Yitzhak Rabin[2], and Hanna Salman[1,*]

[1] Department of Physics and Astronomy, University of Pittsburgh, Pittsburgh, PA, USA
[2] Department of Physics, Bar-Ilan University, Ramat-Gan, Israel
[♯] Equal contribution
[†] Current address: Department of Molecular, Cellular and Developmental Biology, Yale University, New Haven, CT, USA
[*] Correspondence should be addressed to: hsalman@pitt.edu

Supplementary Methods

Estimating mRNAs ratio using quantitative RT-PCR

During the PCR process, the amount of amplified DNA of each target mRNA was measured by fluorescence at the end of each amplification cycle. The ratio of *minE* mRNA to *minD* mRNA was then calculated as follows:
The target amplification during a PCR process follows:

$$X_n = X_0 \cdot 2^n$$

Here,
X_n is the number of target molecules during cycle n
X_0 is the initial number of target molecules
For calculating the ratio of mRNAs of two target genes A and B, we take the threshold number of target molecules to be reached by both genes to be X_T and find the number of cycles (n_A and n_B) needed for each gene to reach that threshold. Then:

$$X_T = X_{0,A}(2)^{n_A}$$
$$X_T = X_{0,B}(2)^{n_B}$$

Here, $X_{0,A}$ and $X_{0,B}$ are the initial numbers of molecules for each of the genes A and B respectively. From the above two equations, we get:

$$X_{0,A}(2)^{n_A} = X_{0,B}(2)^{n_B}$$

And therefore:

$$ratio = \frac{X_{0,A}}{X_{0,B}} = 2^{n_B - n_A}$$

1-D simulation of MinCDE oscillations

The pole-to-pole oscillations were simulated using the exact equations described in Huang et al[1], but reduced to 1-D. In short, the equations are:

$$\frac{\partial \rho_{D:ADP}}{\partial t} = D_D \nabla^2 \rho_{D:ADP} - \sigma_D^{ADP \to ATP} \rho_{D:ADP} + \sigma_{de}\rho_{de}$$

$$\frac{\partial \rho_{D:ATP}}{\partial t} = D_D \nabla^2 \rho_{D:ATP} + \sigma_D^{ADP \to ATP} \rho_{D:ADP} - [\sigma_D + \sigma_{dD}(\rho_d + \rho_{de})]\rho_{D:ATP}$$

$$\frac{\partial \rho_E}{\partial t} = D_E \nabla^2 \rho_E + \sigma_{de}\rho_{de} - \sigma_E \rho_d \rho_E$$

$$\frac{\partial \rho_d}{\partial t} = -\sigma_E \rho_d \rho_E + [\sigma_D + \sigma_{dD}(\rho_d + \rho_{de})]\rho_{D:ATP}$$

$$\frac{\partial \rho_{de}}{\partial t} = -\sigma_{de}\rho_{de} + \sigma_E \rho_d \rho_E$$

Where $\rho_{D:ADP}$, $\rho_{D:ATP}$, and ρ_E are the concentrations in the cytoplasm of MinD:ADP complexes, MinD:ATP complexes, and MinE, and ρ_d, and ρ_{de} are the concentrations on the membrane of MinD:ATP complexes and MinE:MinD:ATP complexes.

The simulations discretizes and solve these equations on a 1D lattice with grid spacing *dx=0.05*µm.

Units of parameters:
Length : µm,
Time: Seconds,
Conc. = number of molecules/µm.

The following values of kinetic parameters were used in all simulations.
Diffusion constants:

$D_D = D_E = 2.5\ \mu m^2/sec$

Reaction rates:

$\sigma_D^{ADP \to ATP} = 1\ sec^{-1}$

$\sigma_D = 0.025\ \mu m/sec$

$\sigma_{dD} = 0.015\ \mu m^3/sec$

$\sigma_{de} = 0.4\ sec^{-1}$

$\sigma_E = 0.93\ \mu m^3/sec$

The initial concentrations of minE and cell length (L) were the only parameters changed between simulations.

Supplementary Figures:

Figure S1

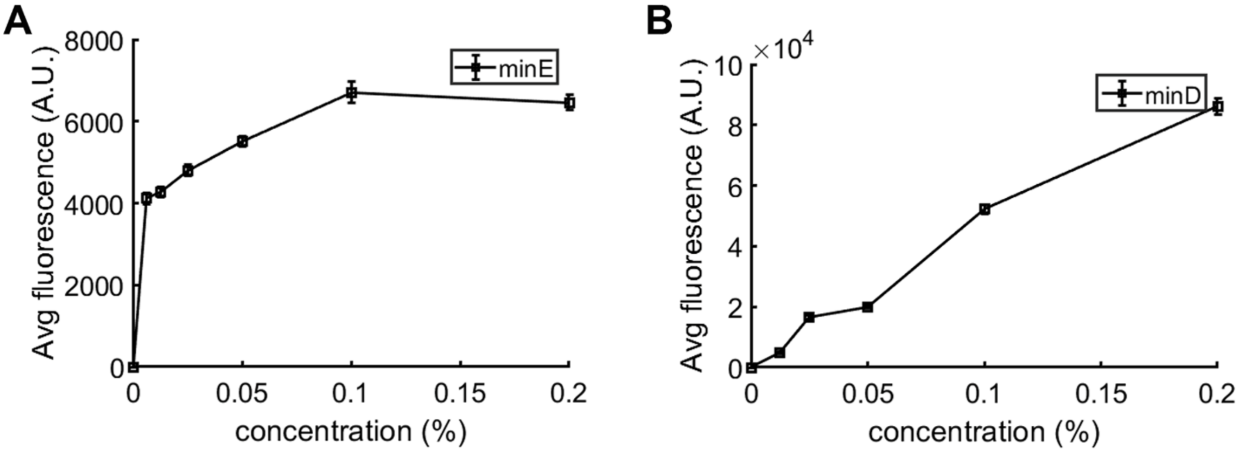

Figure S1. Overexpression of MinE and MinD as a function of arabinose concentration. The average total fluorescence of MinE-mEos (A) and mEos-MinD (B) is measured in cells induced with different concentrations of arabinose as indicated on the x-axes, measured in unites of % w/v. Cell cultures were prepared and induced as described in Materials and Methods subsection "Population level measurements of MinE/MinD effect on cell size". Samples of each culture were then collected and fluorescence images of the 500 cells were acquired to evaluate the overexpression level of both MinE-mEos and mEos-MinD. Error bars depict the standard deviation of all measurements. Source data are provided as a Source Data file.

Figure S2

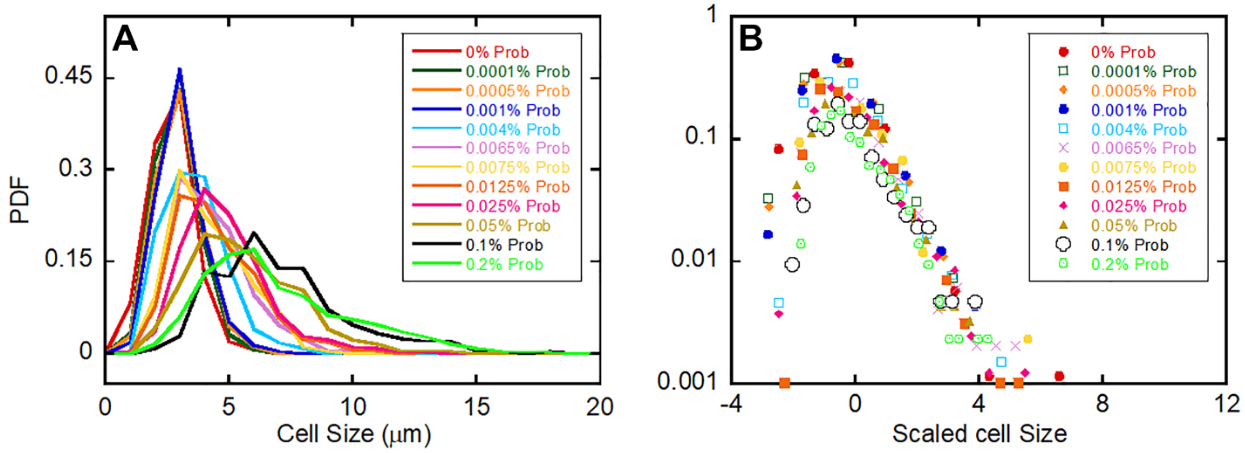

Figure S2. Cell size distributions for various levels of *minE* overexpression. Images of cells in which the expression of *minE-mEos* was induced at various levels of arabinose as indicated in the figure legend, were acquired and the size of several hundreds of cells was measured. (A) depicts the size distribution for the different levels of inductions. (B) The size for each condition measurement was scaled by subtracting the average size of the population in each condition and dividing by standard deviation. Our results show that the scaling of the size distributions reported previously[2] is maintained here as well. Source data are provided as a Source Data file.

Figure S3

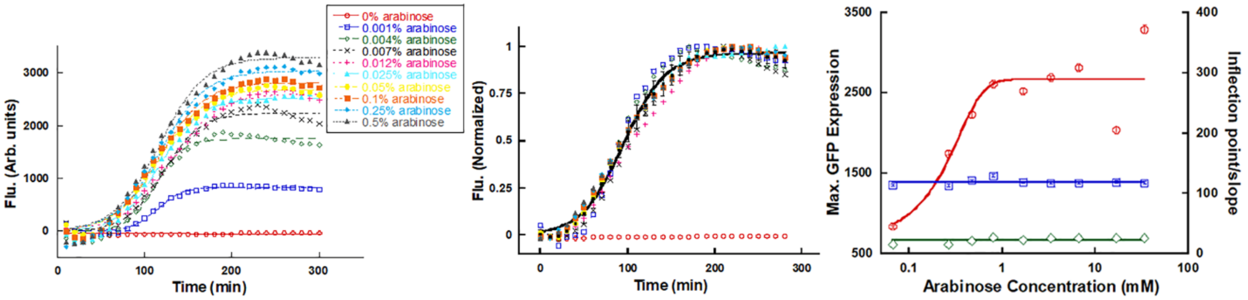

Figure S3. Expression kinetics of the P_{araBAD} promoter. The *gfp* gene was placed under the control of P_{araBAD}, and the fluorescence intensity of GFP was measured as a function of time following the addition of arabinose to the growth medium to induce its expression. The measurements were carried out using the Tecan Infinite M200 microplate reader, while the cells were growing in 96-well plates following the induction of *gfp* expression. Measurements of GFP fluorescence intensity were acquired every 5 minutes together with the OD_{600nm}, and the fluorescence measurement was normalized by the OD_{600nm} measurement to extract the average single-cell fluorescence intensity. (left panel) The fluorescence intensity kinetics following the gfp expression induction by different concentrations of arabinose as indicated in the figure legend. Each data set represent the average of three measurements of the same conditions carried out simultaneously in three different wells. The lines in the graph depict the best fit of:

$$Flu(t) = \frac{Flu_{max}}{\left(1 + e^{-\frac{t-t_c}{t_s}}\right)}$$

where Flu_{max} is the maximal fluorescence, t_c (the inflection point) is the time at which the fluorescence reaches half of its maximal value, and t_s (the slope) is the time after t_c required for the fluorescence to reach two thirds of its maximal value. The values of Flu_{max}, t_c, and t_s for all the arabinose concentrations are depicted in the right panel graph. As it is clear there, t_c and t_s are not affected by the arabinose concentration, and therefore, once normalized, all the data points could be fit to one function as depicted in the middle panel. This fit:

$$Flu(t) = \frac{0.965 \pm 0.008}{\left(1 + e^{-\frac{t-(95.045 \pm 1.12 min)}{24.32 \pm 0.96 min}}\right)}$$

was used to calculate the MinE expression kinetics in the Results subsection Experimental results confirm model predictions. The Flu_{max} as a function of arabinose concentration is depicted by the function:

$$Flu_{max}(C) = \frac{2666.1 \mp 157.07}{\left(1 + e^{-\frac{C-(0.182 \mp 0.07\ mM)}{0.158 \mp 0.077\ mM}}\right)}$$

Source data are provided as a Source Data file.

Figure S4

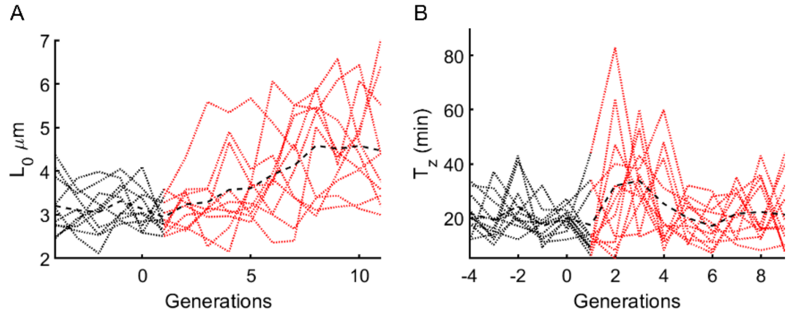

Figure S4. Cell size and FtsZ ring kinetics following induction of MinE overexpression. (A) Example measurements of cell size at birth as a function of generation number, where generation zero, is when the overexpression of *minE-mEos* was induced as described in the Material and Methods subsection "Single-cell level measurements of cell size and FtsZ ring formation dynamics". Each line depict a single-cell level measurement of a lineage in the mother machine. (B) The same as (A) for the time from cell birth required for the FtsZ-mVenus to accumulate to 65% of its maximal fluorescence intensity at mid-cell. Collection of several traces of cell size at birth during the transition, with enough data points before and after the induction of minE overexpression. Source data are provided as a Source Data file.

Figure S5

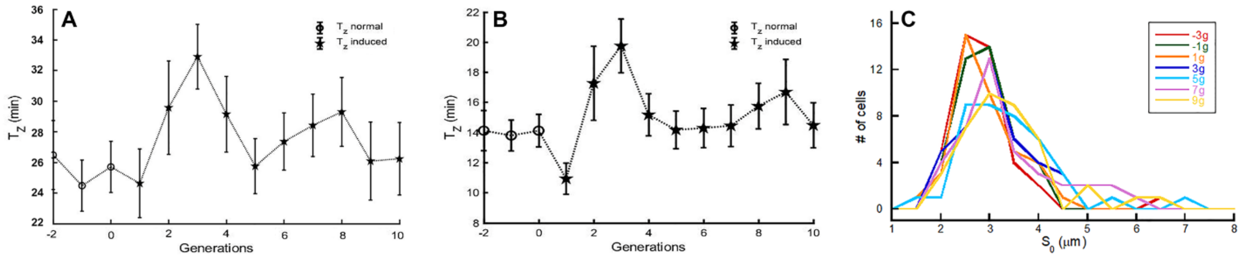

Figure S5. T_z and distribution of cell size at birth during induction of *minE* overexpression. (A and B) Time to reach 75% and 45% of maximal FtsZ ring intensity, respectively, during the induction of *minE* overexpression. The dynamics of Tz in both cases resembles that presented in Fig.4B. (C) The distributions of birth sizes at different time points during the induction process of *minE* overexpression are presented as indicated in the legend, where 1g is the first division event following the addition of the inducer (arabinose) to the growth medium. The averages of these measurements as a function of time are presented in Fig. 4G. Source data are provided as a Source Data file.

Figure S6

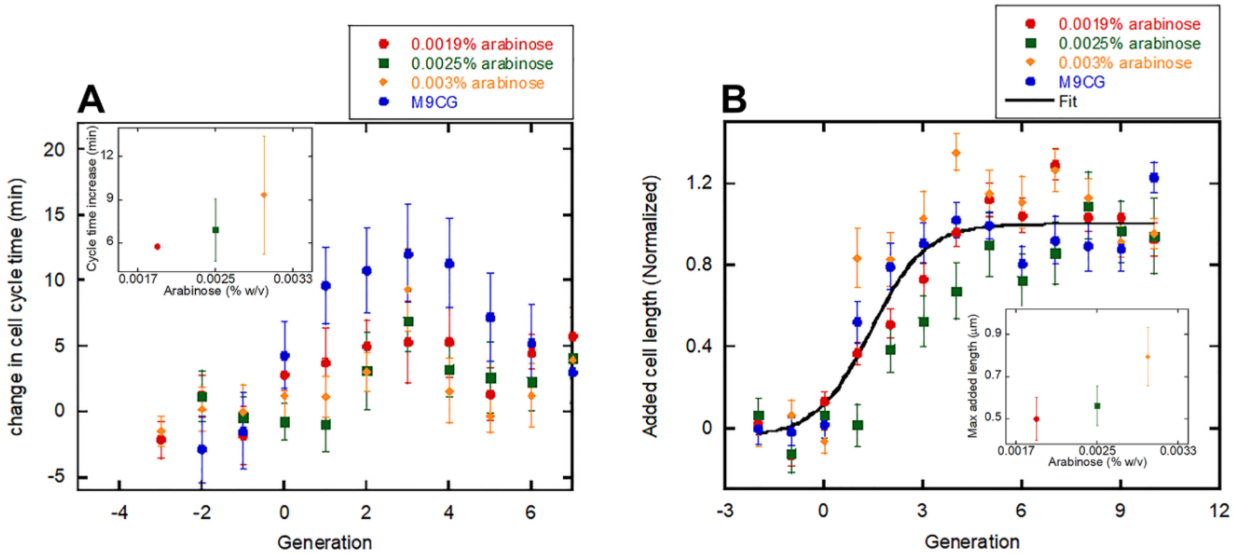

Figure S6. Comparison of added length and cell cycle time under different conditions. (A) Cell cycle duration following the induction of *minE* overexpression with different levels of inducer as indicated in the legend. All conditions show the same transient behavior, albeit the increase in cell cycle time appears to scale with the induction level (inset: maximal increase in cell cycle time measured during the induction process for the different induction levels). This is due to the fact that producing more *minE* requires a longer cell to allow stable FtsZ ring formation, and therefore longer time of growth to reach that length. (B) Cell size at birth following the induction of *minE* overexpression with different levels of inducer and in a different medium (M9CG, in which the average doubling time is ~50 minutes). The normalized size ($\frac{S_0-\langle S_0\rangle_{uninduced}}{\langle S_0\rangle_{induced-final}-\langle S_0\rangle_{uninduced}}$) increase exhibit similar dynamics regardless of the experimental conditions. This is to be expected, because the induction dynamics of the P_{araBAD} promoter is the same for all induction levels (see section of model predictions and Fig. S3). However, the inset shows that the final stable cell length ($\langle S_0\rangle_{induced-final}$) scales with the induction level as projected by the model. The line is intended to show the dynamics trend. It depicts a sigmoidal fit to the data with an exponential rate of increase of 0.83 generation (24.8 minutes), which is the same as obtained from the fluorescence measurements presented in Fig. S9. Each point in the graphs represent the average of 20 – 40 single-cell measurements (0.0019% and 0.003% 20 cells each, 0.002% and M9CG 40 cells each), and the error bars represent the standard error. Source data are provided as a Source Data file.

Figure S7

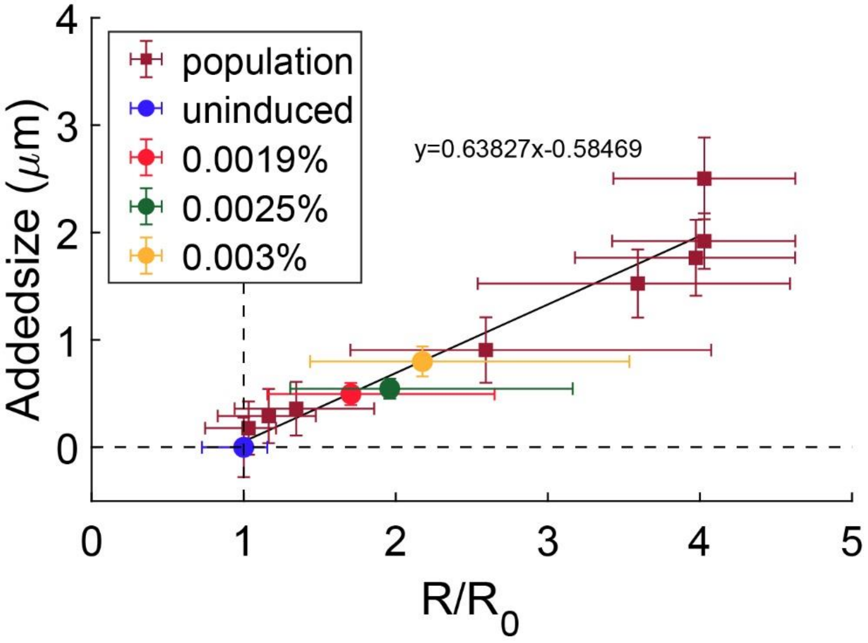

Figure S7. Added size as a function of the increase in the MinE/MinD ratio relative to the uninduced ratio. The graph depicts the increase in cell size as a function of the change in the ratio MinE/MinD obtained from population (Fig. 2) and single-cell measurements (Fig. S6) under different induction levels of minE. Note that while the increase in size is not strictly linear, it can be well-fitted by a linear function within the range of induction tested in the single-cell experiments. More importantly, both population and single-cell measurements of the increase in average cell size agree with each other. Source data are provided as a Source Data file.

Figure S8

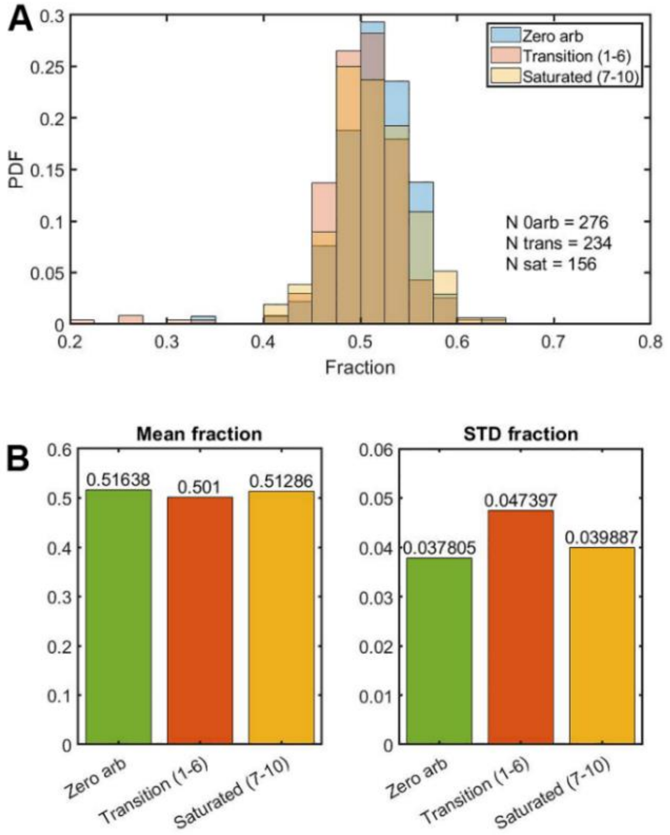

Figure S8. The effect of cell elongation on the division symmetry. (A) Presents the distribution of fractions cells receive from their mother upon division during elongation from one steady state to another measured in the single cell experiments carried out in the mother machine. The mean and standard deviation of these distributions are presented in (B). These results show that the division remains symmetric throughout the transition and after reaching the final steady state just as before the induction of *minE* overexpression. However, the standard deviation is increased during the transition. Source data are provided as a Source Data file.

Figure S9

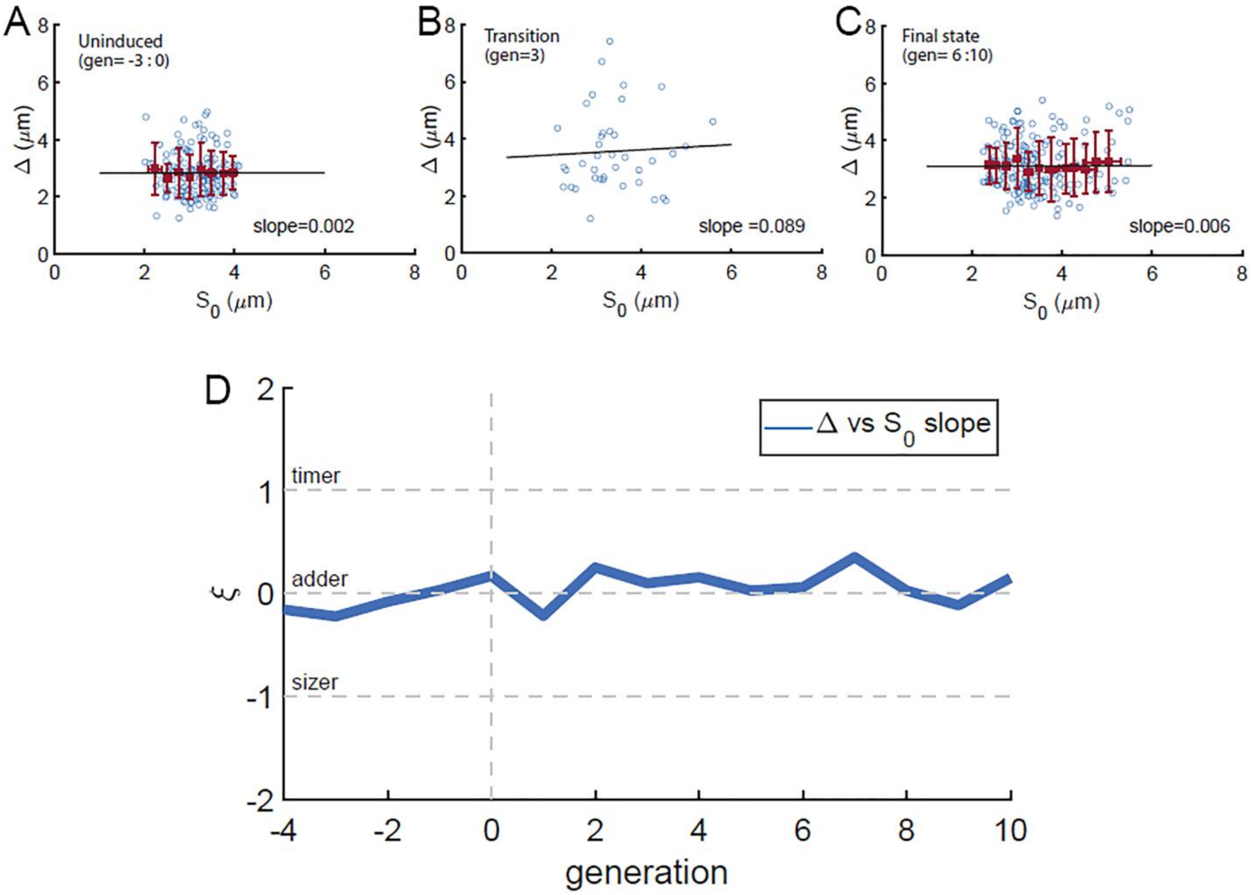

Figure S9. The adder test. The added size (Δ) as a function of birth size (S_0) measured in the 4 generations prior to induction (140 points total, A), in generation 3 following the induction, which is in the middle of the transition (39 points total, B), and in the last 4 generations after the cells have reached the new steady state following the induction of *minE* over expression (140 points total, C). The red squares in (A) and (C) represent the average of the binned data, and the error bars are the standard deviation of the data points in each bin. The slope of Δ as a function of S_0 was also determined for each generation separately throughout the induction process of *minE* overexpression. The results presented in (D) show that the slope remains almost constant and close to zero throughout the transition from one steady state to another. The data presented in this figure are taken from the single cell measurements summarized in Fig. 4, which include 39 cells. Source data are provided as a Source Data file.

Figure S10

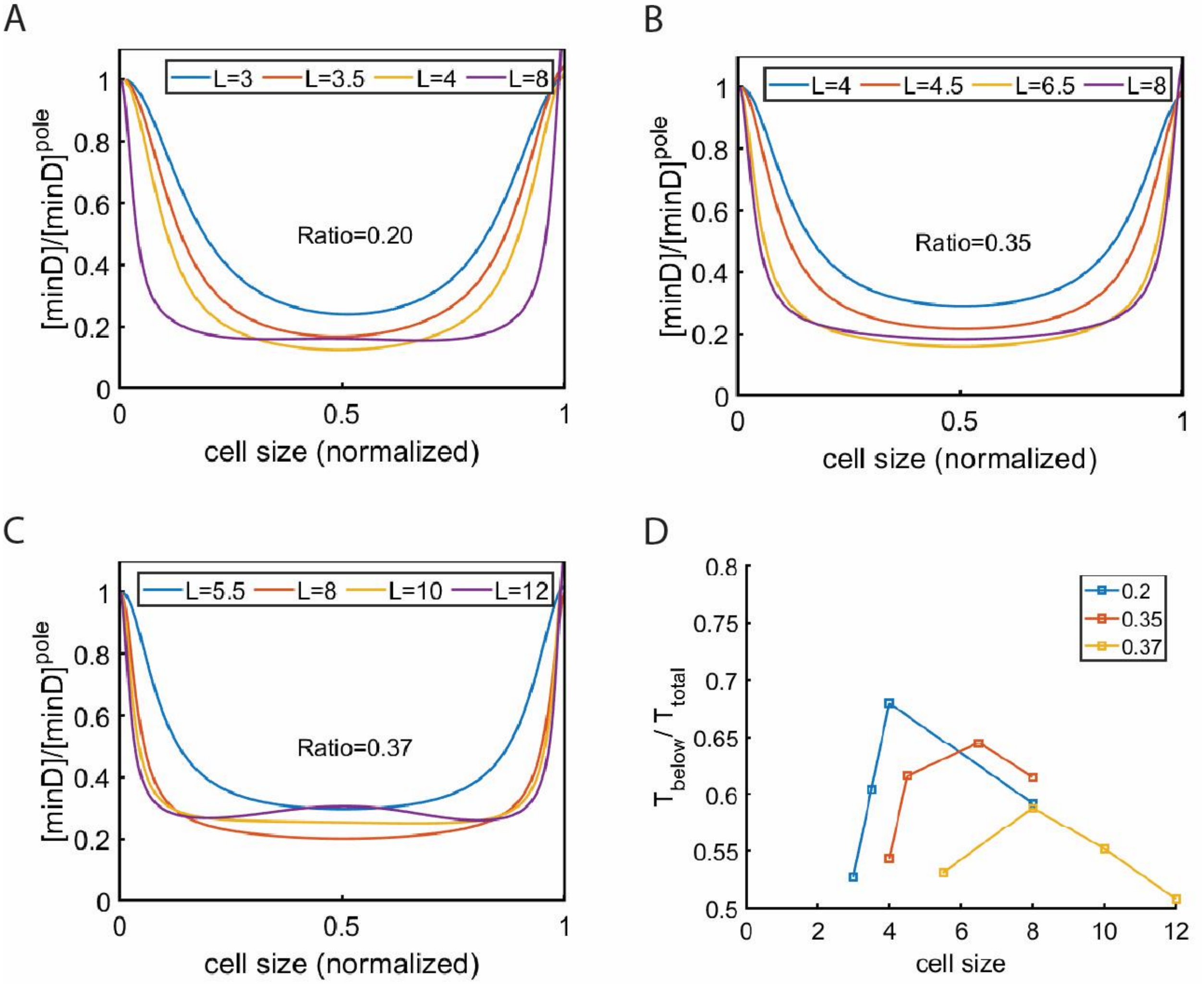

Figure S10. Simulations Results. Simulations of the Min poteints oscillations were carried out as described earlier in SI. Regular pole to pole oscillations are not observed below certain cell lengths, which depends on the ratio of the MinE/MinD proteins in the cell. The average profiles of MinD concentration along the cell normalized by the MinD concentration at the poles for different MinE/MinD ratios and different cell lengths are presented in (A – C). The different color lines represent the profiles in cells of different lengths as indicated in the legend of each figure. In all figures, the x-axis, representing the position along the cell length, has been normalized such that the poles are zero and one. For each ratio tested, we take the first cell length at which the regular pole-to-pole oscillations are observed and determine the average MinD surface concentration at mid-cell (D_{avg}). We then calculate the ratio of the amount of time mid-cell MinD surface concentration is below the average (T_{below}) to the total time (T_{total}). At this length, this ratio is ~1/2 indicating regular oscillations. The length is then changed, a new simulation is carried out and the ratio T_{below}/T_{total} is calculated. This ratio reflects the probability of FtsZ binding at mid-cell, and therefore, we use it to determine the optimal cell length, for which this ratio is maximal. This process is repeated for different MinE/MinD ratios, which are achieved by changing MinE concentration in the simulations. Note that the choice of the reference threshold to calculate T_{below}, which we took to be D_{avg}, does not alter the results and we still get the same optimum length that maximizes the ratio of the dwell times, which are presented in (D). The different colors represent the results for different MinE/MinD ratio. Note that for each ratio, the time fraction during which the mid-cell is free of MinD maximizes at a different cell length. Source data are provided as a Source Data file.

Figure S11

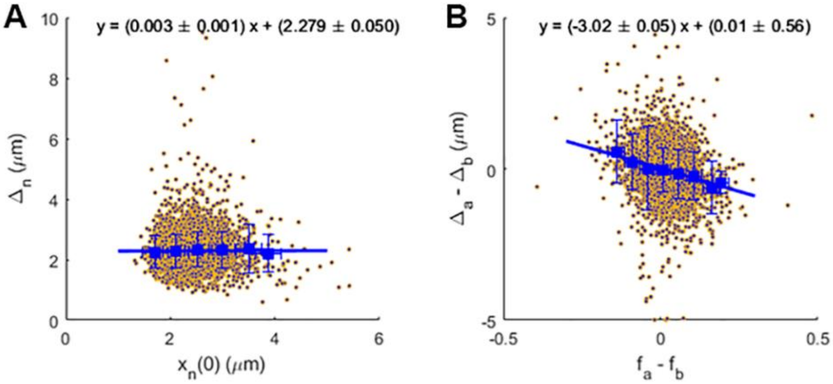

Figure S11. Comparison between sisters added size. The added size measured in previous experiments[3] was used to verify that the smaller sister was indeed adding larger size compared to its larger sibling. (A) shows that our data (8,173 cells) agrees with the adder phenomenon, where the size added during each cell cycle is independent of the size of the cell at the start of the cycle. Nevertheless, when comparing the added size of sisters, we find that the added size difference between sisters ($\Delta_a - \Delta_b$) (3,942 sisters) is negatively correlated with the difference between the fractions ($f_a - f_b$) each sister received from the mother (B). The blue squares in both graphs represent the average of the binned data, and the error bars are the standard deviation of the data points in each bin. The data of this figure is taken from[3]. Source data are provided as a Source Data file.

Supplementary References:

1. Huang, K. C., Meir, Y. & Wingreen, N. S. Dynamic structures in Escherichia coli: spontaneous formation of MinE rings and MinD polar zones. *Proc. Natl. Acad. Sci. U. S. A.* **100**, 12724–8 (2003).
2. Salman, H. *et al.* Universal protein fluctuations in populations of microorganisms. *Phys. Rev. Lett.* **108**, 238105 (2012).
3. Kohram, M., Vashistha, H., Leibler, S., Xue, B. K. & Salman, H. Bacterial Growth Control Mechanisms Inferred from Multivariate Statistical Analysis of Single-Cell Measurements. *Curr. Biol.* (2021) doi:10.1016/j.cub.2020.11.063.
