# Supplementary material for: Bacterial cell-size changes resulting from altering the relative expression of Min proteins
Source: Nat Commun. 2023 Sep 15;14:5710. doi: 10.1038/s41467-023-41487-0 (PMC10504268; doi:10.1038/s41467-023-41487-0)
Supplement: Supplementary file 3 — Description of Additional Supplementary Files [file 41467_2023_41487_MOESM3_ESM.pdf]

Description of Additional Supplementary Files:

Supplementary Movie 1: A movie of a growth channel, taken at rate of one frame per minute in a typical experiment, where the FtsZ ring is visible in green. The cell is also expressing mCherry to allow better detection of its boundary. The cell size dynamics and the accumulation kinetics of FtsZ at the septum, presented in Fig. 4, were evaluated from this and similar movies.

Supplementary Movie 2: A movie of a simulation result showing the oscillation dynamics of the MinCD (green) and MinE (purple) in a 4µm long cell with the optimal ratio of MinD/MinE.

Supplementary Movie 3: The same as video Supplementary Movie 2 with a slightly higher MinD/MinE ratio. Notice that the oscillation frequency is now larger, which lead to larger occupation time of the cell membrane at the middle of the cell.

Supplementary Software: Code used to carry out the 1-D simulations of the Min oscillations as described in the main text and Supplementary Information.
