# Supplementary material for: Bacterial cell-size changes resulting from altering the relative expression of Min proteins
Source: Nat Commun. 2023 Sep 15;14:5710. doi: 10.1038/s41467-023-41487-0 (PMC10504268; doi:10.1038/s41467-023-41487-0)
Supplement: Supplementary file 7 — Supplementary software [file 41467_2023_41487_MOESM7_ESM.zip › Vashistha_Supplementary Software.docx]

**Supplementary Software**

Bacterial cell-size changes resulting from altering the relative expression of Min proteins

Harsh Vashistha^1,♯,†^, Joanna Jammal-Touma^1,♯^, Kulveer Singh^2^, Yitzhak Rabin^2^, and Hanna Salman^1,*^

^1^ Department of Physics and Astronomy, University of Pittsburgh, Pittsburgh, PA, USA

^2^ Department of Physics, Bar-Ilan University, Ramat-Gan, Israel

^♯^ Equal contribution

^†^ Current address: Department of Molecular, Cellular and Developmental Biology, Yale University, New Haven, CT, USA

^*^ Correspondence should be addressed to: [hsalman@pitt.edu](mailto:hsalman@pitt.edu)

// 1D code for MinD-MinE oscillations (Huang et al equations)in c++

#include <fstream>

#include <iostream>

#include <exception>

#include <cstdio>

#include <cstdlib>

#include <cmath>

#include <time.h>

#include <string>

#include <iomanip>

#include <vector>

#include <complex>

#include <stdbool.h>

#include<random>

#include <chrono>

**using** **namespace** std**;**

#define PI 3.14159265

double dx**=**0.05**;**

//to caculate laplacian

double lap**(**int index**,** int xSteps**,** double c0**,** double c1**,** double c2**)**

**{**

//computing node is c1

double cx**;**

**if(**index**==**1**)**

**{**

cx**=(**c2**-**c1**)/(**dx*****dx**);**

**}else** **if(**index**==**xSteps**+**1**)**

**{**

cx**=(**c0**-**c1**)/(**dx*****dx**);**

**}else**

**{**

cx**=(**c0**-**2*****c1**+**c2**)/(**dx*****dx**);**

**}**

**return** cx**;**

**}**

int main **(**int argc**,** char *****argv**[]** **)**

**{**

//input lambda, sigma, omega, time of run

**if** **(**argc **<** 4**)**

**{**

std**::**cerr **<<** "Usage: " **<<** argv**[**0**]** **<<**" L"**<<**" minD_rho"**<<**" minE_rho"**<<**std**::**endl**;**

**return** 1**;**

**}**

//file to store all proteins separately at different times

ofstream ReadFile**;**

char Filename**[**100**];**

sprintf**(**Filename**,** "data_L_%f_minD_%f_minE_%f.dat"**,** atof**(**argv**[**1**]),** atof**(**argv**[**2**]),** atof**(**argv**[**3**]));**

ReadFile**.**open**(**Filename**);**

//file to store total minD and minE in entire cell (to check conservation)

ofstream ReadFile1**;**

char Filename1**[**100**];**

sprintf**(**Filename1**,** "total_L_%f_minD_%f_minE_%f.dat"**,** atof**(**argv**[**1**]),** atof**(**argv**[**2**]),** atof**(**argv**[**3**]));**

ReadFile1**.**open**(**Filename1**);**

//file to store protein (total (eg. minD_ADP+minDATP) in bulk and on surface) at different x

ofstream ReadFile2**;**

char Filename2**[**100**];**

sprintf**(**Filename2**,** "data_total_L_%f_minD_%f_minE_%f.dat"**,** atof**(**argv**[**1**]),** atof**(**argv**[**2**]),** atof**(**argv**[**3**]));**

ReadFile2**.**open**(**Filename2**);**

// file to store time average value of proteins at different x

ofstream ReadFile3**;**

char Filename3**[**100**];**

sprintf**(**Filename3**,** "data_avg_L_%f_minD_%f_minE_%f.dat"**,** atof**(**argv**[**1**]),** atof**(**argv**[**2**]),** atof**(**argv**[**3**]));**

ReadFile3**.**open**(**Filename3**);**

//parameters. Length is in micrometer, time in sec, conc. = # of molecule/micrometer

double sg_D_ADP_ATP**,** sg_de**,** sg_D**,** sg_dD**,** sg_E**,** D_D**,** D_E**;**

D_D**=**2.5**;**

D_E**=**2.5**;**

sg_D_ADP_ATP**=**1.0**;**

sg_D**=**0.025**;**

sg_dD**=**0.0015*****10**;**

sg_de**=**0.4**;**

sg_E**=**0.093*****10**;**

//average concentration of minD, minE in number of molecule per micrometer

double ro_D_initial**,** ro_E_initial**;**

ro_D_initial**=**atof**(**argv**[**2**]);**

ro_E_initial**=**atof**(**argv**[**3**]);**

double D**,** L**,** T**,** dt**;**

L **=** atof**(**argv**[**1**]);** //Length of bacteria

T**=**1000.0**;** //Time in seconds

dt **=** 0.0001**;**//timestep size

int xSteps**,** tSteps**;**

xSteps**=**int**(**L**/**dx**);**//number of lattice points

tSteps**=**int**(**T**/**dt**)+**1**;**// Number of timesteps

//array of density at all lattice points

double ro_D_ADP0**[**xSteps**+**3**],** ro_D_ADP1**[**xSteps**+**3**],** ro_E0**[**xSteps**+**3**],** ro_E1**[**xSteps**+**3**],** ro_D_ATP0**[**xSteps**+**3**],** ro_D_ATP1**[**xSteps**+**3**],** ro_d0**[**xSteps**+**3**],** ro_d1**[**xSteps**+**3**],** ro_de0**[**xSteps**+**3**],** ro_de1**[**xSteps**+**3**];**

//double c_x, c_initial=100.0;

//Initializing the concentration at lattice points

**for(**int j**=**1**;** j**<=**xSteps**+**1**;**j**++)**

**{**

ro_D_ADP0**[**j**]=(-**j*****ro_D_initial*****L*****2**/**double**((**xSteps**)*(**xSteps**+**1**))** **+** ro_D_initial*****L*****2**/**double**(**xSteps**));**

ro_E0**[**j**]=(-**j*****ro_E_initial*****L*****2**/**double**((**xSteps**)*(**xSteps**+**1**))** **+** ro_E_initial*****L*****2**/**double**(**xSteps**));**

ro_D_ATP0**[**j**]=**0.0**;**

ro_d0**[**j**]=**0.0**;**

ro_d1**[**j**]=**0.0**;**

**}**

//concentration at redundant lattice points

ro_D_ADP0**[**0**]=**0.0**;**

ro_D_ADP0**[**xSteps**+**2**]=**0.0**;**

ro_E0**[**0**]=**0.0**;**

ro_E0**[**xSteps**+**2**]=**0.0**;**

ro_D_ATP0**[**0**]=**0.0**;**

ro_D_ATP0**[**xSteps**+**2**]=**0.0**;**

ReadFile**<<**"#"**<<**"x "**<<**"minD_ADP "**<<**"minE "**<<**"minD_ATP "**<<**"mind "**<<**"minde "**<<**endl**;**

ReadFile2**<<**"#"**<<**"minD_total "**<<**"minE_total "**<<**"minD_surface "**<<**"minE_surface"**<<**endl**;**

// checking the initial distribution and total molecule conservation and writing in files

double total_D**=**0.0**,** total_E**=**0.0**,** minD_total**,** minE_total**,** minD_surface**,** minE_surface**;**

**for(**int j**=**1**;** j**<=**xSteps**+**1**;**j**++)**

**{**

total_D**+=**ro_D_ADP0**[**j**]+**ro_D_ATP0**[**j**]+**ro_d0**[**j**]+**ro_de0**[**j**];**

total_E**+=**ro_E0**[**j**]+**ro_de0**[**j**];**

minD_total**=**ro_D_ADP0**[**j**]+**ro_D_ATP0**[**j**]+**ro_d0**[**j**]+**ro_de0**[**j**];**

minE_total**=**ro_E0**[**j**]+**ro_de0**[**j**];**

minD_surface**=**ro_d0**[**j**]+**ro_de0**[**j**];**

minE_surface**=**ro_de0**[**j**];**

ReadFile**<<**dx*****j**<<**' '**<<**ro_D_ADP0**[**j**]<<**' '**<<**ro_E0**[**j**]<<**' '**<<**ro_D_ATP0**[**j**]<<**' '**<<**ro_d0**[**j**]<<**' '**<<**ro_de0**[**j**]<<**endl**;**

ReadFile2**<<**dx*****j**<<**' '**<<**minD_total**<<**' '**<<**minE_total**<<**' '**<<**minD_surface**<<**' '**<<**minE_surface**<<**endl**;**

**}**

ReadFile**<<**" "**<<**endl**;**

ReadFile**<<**" "**<<**endl**;**

ReadFile**<<**" "**<<**endl**;**

ReadFile2**<<**" "**<<**endl**;**

ReadFile2**<<**" "**<<**endl**;**

ReadFile2**<<**" "**<<**endl**;**

ReadFile1**<<**0**<<**' '**<<**total_D**<<**' '**<<**total_E**<<**endl**;**

//array to compute average value of proteins at different lattice points

double avg_minD_total**[**xSteps**+**2**]** **={**0.0**},** avg_minE_total**[**xSteps**+**2**]={**0.0**},** avg_minD_surface**[**xSteps**+**2**]={**0.0**},** avg_minE_surface**[**xSteps**+**2**]={**0.0**};**

int count **=**0**;**

double free_time_minD**[**xSteps**+**2**]={**0.0**},** free_time_minE**[**xSteps**+**2**]={**0.0**};**

**for(**int i **=** 1**;** i**<**tSteps**;** i**++)**

**{**

**for(**int j**=**1**;** j**<=**xSteps**+**1**;** j**++)**

**{**

//Huang et al. reaction diffusion equations

ro_D_ADP1**[**j**]** **=** ro_D_ADP0**[**j**]** **+** dt*****D_D*****lap**(**j**,** xSteps**,** ro_D_ADP0**[**j**-**1**],** ro_D_ADP0**[**j**],** ro_D_ADP0**[**j**+**1**])** **-** dt*****sg_D_ADP_ATP*****ro_D_ADP0**[**j**]** **+** dt*****sg_de*****ro_de0**[**j**];**

ro_E1**[**j**]** **=** ro_E0**[**j**]** **+** dt*****D_E*****lap**(**j**,** xSteps**,** ro_E0**[**j**-**1**],** ro_E0**[**j**],** ro_E0**[**j**+**1**])** **-** dt*****sg_E*****ro_d0**[**j**]***ro_E0**[**j**]** **+** dt*****sg_de*****ro_de0**[**j**];**

ro_D_ATP1**[**j**]** **=** ro_D_ATP0**[**j**]** **+** dt*****D_D*****lap**(**j**,** xSteps**,** ro_D_ATP0**[**j**-**1**],** ro_D_ATP0**[**j**],** ro_D_ATP0**[**j**+**1**])** **+** dt*****sg_D_ADP_ATP*****ro_D_ADP0**[**j**]** **-** dt*****sg_D*****ro_D_ATP0**[**j**]** **-** dt*****sg_dD***(**ro_d0**[**j**]+**ro_de0**[**j**])***ro_D_ATP0**[**j**];**

ro_d1**[**j**]=**ro_d0**[**j**]** **+** dt*****sg_D*****ro_D_ATP0**[**j**]** **-** dt*****sg_E*****ro_d0**[**j**]***ro_E0**[**j**]** **+** dt*****sg_dD***(**ro_d0**[**j**]+**ro_de0**[**j**])***ro_D_ATP0**[**j**];**

ro_de1**[**j**]=**ro_de0**[**j**]** **+** dt*****sg_E*****ro_d0**[**j**]***ro_E0**[**j**]** **-** dt*****sg_de*****ro_de0**[**j**];**

**}**

//cout<<"###########"<<endl;

**for(**int j**=**1**;** j**<=**xSteps**+**1**;** j**++)**

**{**

ro_D_ADP0**[**j**]=**ro_D_ADP1**[**j**];**

ro_E0**[**j**]=**ro_E1**[**j**];**

ro_D_ATP0**[**j**]=**ro_D_ATP1**[**j**];**

ro_d0**[**j**]=**ro_d1**[**j**];**

ro_de0**[**j**]=**ro_de1**[**j**];**

**}**

total_D**=**0.0**;**

total_E**=**0.0**;**

**if(**i**%**10000**==**0**)**

**{**

cout**<<**i**/**10000**<<**endl**;**

**for(**int j**=**1**;** j**<=**xSteps**+**1**;** j**++)**

**{**

total_D**+=**ro_D_ADP0**[**j**]+**ro_D_ATP0**[**j**]+**ro_d0**[**j**]+**ro_de0**[**j**];**

total_E**+=**ro_E0**[**j**]+**ro_de0**[**j**];**

minD_total**=**ro_D_ADP0**[**j**]+**ro_D_ATP0**[**j**]+**ro_d0**[**j**]+**ro_de0**[**j**];**

minE_total**=**ro_E0**[**j**]+**ro_de0**[**j**];**

minD_surface**=**ro_d0**[**j**]+**ro_de0**[**j**];**

minE_surface**=**ro_de0**[**j**];**

ReadFile**<<**dx*****j**<<**' '**<<**ro_D_ADP0**[**j**]<<**' '**<<**ro_E0**[**j**]<<**' '**<<**ro_D_ATP0**[**j**]<<**' '**<<**ro_d0**[**j**]<<**' '**<<**ro_de0**[**j**]<<**endl**;**

ReadFile2**<<**dx*****j**<<**' '**<<**minD_total**<<**' '**<<**minE_total**<<**' '**<<**minD_surface**<<**' '**<<**minE_surface**<<**endl**;**

**if(**count**>**10000**)**

**{**

avg_minD_total**[**j**]+=**minD_total**;**

avg_minE_total**[**j**]+=**minE_total**;**

avg_minD_surface**[**j**]+=**minD_surface**;**

avg_minE_surface**[**j**]+=**minE_surface**;**

**}**

**}**

count**++;**

//storing total protein (to check conservation)

ReadFile1**<<**i**<<**' '**<<**total_D**<<**' '**<<**total_E**<<**' '**<<**total_D**+**total_E**<<**endl**;**

//cout<<endl;

ReadFile**<<**" "**<<**endl**;**

ReadFile**<<**" "**<<**endl**;**

ReadFile**<<**" "**<<**endl**;**

ReadFile2**<<**" "**<<**endl**;**

ReadFile2**<<**" "**<<**endl**;**

ReadFile2**<<**" "**<<**endl**;**

**}**

**}**

ReadFile3**<<**"#x"**<<**"minD_total "**<<**"minE_total "**<<**"minD_surface "**<<**"minE_surface"**<<**endl**;**

**for(**int j**=**1**;** j**<=**xSteps**+**1**;** j**++)**

**{**

//storing time average at different x

ReadFile3**<<**dx*****j**<<**' '**<<**avg_minD_total**[**j**]/**double**(**count**-**10000**)<<**' '**<<**avg_minE_total**[**j**]/(**count**-**10000**)<<**' '**<<**avg_minD_surface**[**j**]/**double**(**count**-**10000**)<<**' '**<<**avg_minE_surface**[**j**]/**double**(**count**-**10000**)<<**endl**;**

**}**

cout**<<**"sD = "**<<**sg_D**<<**' '**<<**"sdD = "**<<**sg_dD**<<**' '**<<**"sde = "**<<**sg_de**<<**' '**<<**"sE ="**<<**sg_E**<<**endl**;**

**return** 0**;**

**}**
